# Urinary retention concomitant with methamphetamine use: a case report

**DOI:** 10.1186/s13256-021-02705-9

**Published:** 2021-04-02

**Authors:** Ayotunde Olumide Ojo, Adesegun Lawrence Ajasa, Rilwan Babatunde Oladipupo, Nicholas Oluwaseyi Aderinto

**Affiliations:** 1 Outpatient Department, Roding Medical Centre, Lagos, Nigeria; 2 Clinical III, Faculty of Clinical Sciences, LAUTECH, Ogbomoso, Nigeria

**Keywords:** Methamphetamine, Urinary retention, Dysuria, Suprapubic fullness, Anorexic, Urodynamic studies

## Abstract

**Background:**

Urinary retention is a condition in which impaired emptying of the bladder results in postvoid residual urine. It can be acute or chronic urinary retention. There have been only scattered case studies that have described urinary retention resulting from methamphetamine use. This case report is aimed at raising awareness about methamphetamine abuse as an important factor in the aetiological considerations when evaluating cases of urinary retention among healthy younger age groups.

**Case presentation:**

We report a patient who had acute urinary retention after brief amphetamine use. A 26-year-old Nigerian man presented at the emergency room on account of an inability to pass urine and lower abdominal pain. Before this incident, the patient reported a recent ingestion of amphetamine to achieve weight reduction and a fit body. A week after use, he started to experience difficulty passing out urine hence necessitating a visit to the emergency department. After a brief assessment, physical examination revealed a man in painful distress with mild suprapubic fullness. He had a successful passage of a urethral catheter for continuous bladder drainage with dramatic improvement in his symptoms. He was subsequently discontinued on methamphetamine use and referred to a urologist for further evaluation.

**Conclusion:**

Most cases of urinary retention are diagnosed clinically and are rarely missed. But because urinary retention is associated with a wide range of aetiological factors, clinicians need to be aware of the effects of certain drugs in the aetiology of urinary retention. In the management of a case of urinary retention in the younger age group, clinicians should enquire about a history of drug use, the drug of particular interest being methamphetamine, and also employ the use of urodynamic studies in the evaluation of such cases.

## Introduction

Methamphetamine is a psychoactive drug primarily used for recreational purposes. It is a well-known anorectic agent. And it is for this reason, among many others, that methamphetamine abuse is increasing to epidemic proportions. A 2003 survey conducted by the United States Department of Health and Human Services found that 2.1 million individuals aged 12 and older had used ecstasy (methamphetamine) during the preceding year, with approximately 1% of young adults aged 18 to 25 having used it within the past 30 days [1]. The mechanism underlying the anorectic response of methamphetamine has been attributed to its inhibitory effect on hypothalamic neuropeptide Y (NPY), an orexigenic peptide in the brain [2] . Aside from its anorexic effect, when methamphetamine is ingested, it increases the release of monoamine and excitatory neurotransmitters in the brain and also delays their metabolism by inhibiting monoamine oxidase. Its more pronounced effects target the norepinephrine and dopamine neurotransmitter systems which result in increased sympathomimetic action. It is this increased sympathomimetic action that culminates in the induction of spinal reflex potentiation of urethral activities leading to sufficient resistance against voiding of urine. Hence, the role of methamphetamine in urinary retention.

This paper presents a case study in which a patient experienced difficulty passing urine after brief methamphetamine use. His symptoms, while they lasted for weeks, were characterized by alternating resolution and recurrence despite the complete cessation of methamphetamine use. While there are other documented causes of urinary retention in literature, the only identifiable aetiology in our patient is the recent methamphetamine use. Hence, it is our opinion that methamphetamine use/abuse should be part of the aetiological considerations and should be excluded in otherwise healthy young patients who present with unexplained urinary retention.

## Case report

A 26-year-old Nigerian man presented to the emergency unit of our facility on account of difficulty urinating and abdominal pain over the past week. He was initially evaluated for urinary tract infection on account of similar symptoms at the general outpatient clinic a week before the emergency unit presentation. At this initial contact, the patient was treated for urinary tract infection empirically using PO Ciprofloxacin 500 mg bd, based on history and examination coupled with complete blood count and urinalysis findings as follows:

*Complete blood count:*

Packed cell volume: 40%

White blood count: 5000

Neutrophils: 40%

Lymphocytes: 59%

Eosinophils: 1%

*Urinalysis:*

Blood: Negative

Colour: Amber and clear

Glucose: Negative

Ketones: Negative

Leucocytes: Negative

Nitrite: Positive

pH: 6.0

Protein: Negative

Specific gravity: 1.030

Urobilinogen: Normal.

The patient presented a week later with acute urinary retention. He denied any history of urethra discharge, previous occurrence of urinary retention, urethra instrumentation, dysuria, or any recurrent urinary tract infection. He had no significant past medical or surgical history. Pertinent findings from social history are in keeping with occasional alcohol use but does not smoke or use any illicit substance. However, he reported recent ingestion of methamphetamine a few days before his symptoms began.

Physical examination findings revealed a young man in mild painful distress secondary to suprapubic discomfort. Vital signs at presentation: Temperature: 36.5 °C, Pulse rate: 78 beats per minute, Blood pressure: 128/66 mmHg.

Neurological examination revealed a conscious, alert and oriented young man. The speech was clear, fluent and coherent. All cranial nerve functions were intact. Gait is steady with normal steps and intact motor, sensory and coordination functions. Other systemic examination findings were essentially normal except for a moderate fullness noticed in the suprapubic region.

He was commenced on continuous bladder drainage via urethral catheterization using a Foley catheter with a dramatic relief of symptoms. The patient, however, found it very strange using a urethra catheter and opted to have it removed after minutes of passage. He also opted for discharge and subsequent follow-up, if need be. A day later, he presented again to the emergency unit with the same symptoms as before and in acute urinary retention. This necessitated the second urethra catheterisation for continuous bladder drainage. He agreed to have the catheter in-situ after adequate counselling on the need to do so.

**Fig. 1:**
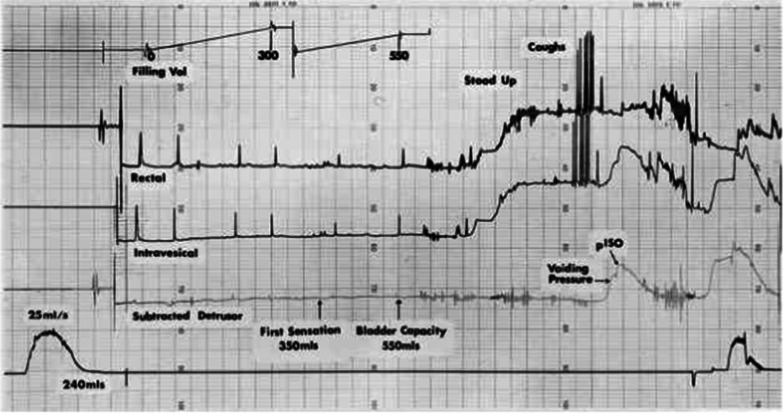
Graphical illustration showing the urodynamic study

Preliminary laboratory and radiological investigations were essentially normal and not suggestive of any genitourinary obstruction. Both abdominal ultrasound (KUB) and abdominal computed tomography scans done revealed an essentially normal urinary tract with preserved corticomedullary differentiation of both kidneys. No renal calculi obstructing urine flow was seen (Fig. 1). However, pertinent findings from an assessment of bladder and sphincteric functions using urodynamics were in keeping with urge incontinence and mild detrusor instability with the voiding cystometrogram showing a high sphincteric pressure and significant bladder contraction.

He was subsequently referred for further evaluation and a urologist's review. Follow up done over the first two months after referral revealed intermittent visits for removal and recatheterization due to alternating resolution and recurrence of symptoms despite the complete cessation of methamphetamine use. He was commenced on α receptor antagonist and antihistamine. Thereafter, he had a gradual improvement in his symptoms and has remained symptom-free for the last 4 months.

## Discussion

While methamphetamine is a central nervous system drug first discovered over a 100 years ago, its availability without prescription has gone into a restricted status as a drug used in the treatment of attention deficit hyperactivity disorder (ADHD) and narcolepsy [3]. Yet, it is the stimulant, euphoric, anorexic, and em-pathogenic properties of the drug that make it a substance widely abused. Methamphetamine, a sympathomimetic amine itself, produces a potent central nervous system (CNS)-mediated stimulant, anorectic, and cardiovascular effects [4]. But for the most part, it is the neurological and behavioural aspects of methamphetamine use that have enjoyed much of focus in the literature. This is understandably so because of methamphetamine’s many properties as a stimulant drug for which its drive as a drug of abuse and popularity is based [5]. This case report is not an effort to emphasize what is already known but to ensure that more focus is directed at the association between methamphetamine abuse and urinary tract symptoms.

It is known that urinary retention can result from any event that increases the resistance to the flow of urine viz mechanical obstruction or dynamic obstruction. The latter is commonly secondary to interruption of either the sensory innervation of the bladder wall or the motor supply of the detrusor muscle or secondary to the influence of drugs [6] . In their review, CH Yee* et al*. [1] illustrated the impact of methamphetamine on the urinary tract specifically the increase in storage symptoms associated with methamphetamine use. The urinary bladder undergoes a repeated cycle of filling and emptying which is influenced by a complex interaction of autonomic nervous control. A loss of this very complex but well-coordinated mechanism of interactions between the bladder, the urethral sphincter, neural pathways, and neurotransmitters impact the act of micturition. This is further confirmed by Koo* et al*. [7] who demonstrated the therapeutic benefits of α blockers and anticholinergics in methamphetamine abusers with lower urinary tract symptoms.

Likewise, chronic methamphetamine consumption was also reported to cause neurogenic bladder and chronic urinary retention. In 2008, Beuerle* et al*. [1] reported a case of chronic methamphetamine use which led to neurogenic bladder and chronic urinary retention in a 21-year-old man. However, the mechanism underlying this, though explained, is unclear. The β-phenylethylamine central structure of methamphetamine allows it to cross the blood-brain barrier easily and makes resistance to degradation that happens in the brain [8]. This structural property acts as a competitor at dopamine’s membrane transporters. The competition at the dopamine membrane transporter leads to enhanced dopamine release from the storage vesicles. These effects cumulate into an increased cytoplasmic dopamine concentration [9]. These dopaminergic pathways, through their sympathomimetic actions, exert an inhibitory effect on voiding by acting through α1 receptor on the internal urethra sphincter. Hence, urinary retention occurs when there is a loss of coordination of the detrusor muscle contraction with the relaxation of the urinary sphincter. All the above findings were evident in the urodynamic studies done in our patient which were in keeping with mild detrusor instability resulting from high sphincteric pressure and significant bladder contraction. Coupled with complete resolution of symptoms only after the use of α receptor antagonist in this patient.

## Conclusion

This paper suggests that methamphetamine use/abuse should be part of the aetiological considerations and should be excluded in otherwise healthy young patients who present with unexplained urinary retention. In the literature analyzed, only a few cases have been reported and the mechanism of urinary retention not clearly put. In view of this, more studies are needed to clarify the mechanism through which urinary retention may occur as a rare but debilitating complication of methamphetamine abuse. Nevertheless, initial efforts should be targeted at the stabilization of patients in terms of relief of symptoms of retention through urethral catheterization and discontinuation of methamphetamine usage in the patient.

## Data Availability

Data sharing not applicable to this article as no datasets were generated or analyzed during the current study.
